# The National Pension Fund

**Published:** 1887-08-06

**Authors:** 


### THE NATIONAL PENSION FUND.

It will save a good deal of trouble if nurses and other hospital officials who propose joining the National Pension Fund will send their names and other particulars to *the Secretary of The Hospitals Association*, Norfolk House, Norfolk Street, W.C., and not to the Editor of The Hospital. Names continue to arrive in considerable numbers, but if those interested desire the Fund to be started before the end of the Jubilee year they must influence and interest all their friends immediately.

### From a Nurse, Hospital for Consumption, Brompton.

Seeing an article this morning in The Hospital for March 26th, I hasten to inform you that I am one, I believe I may say of many trained nurses who would gladly sacrifice some of even their small earnings towards a small yearly income, and if possible a little assistance in time of sickness, or being temporarily disabled from work from accident, or in fact any cause not brought through any fault or misconduct on their part. If you carry out your intention of starting a *bonâ fide* Fund of this kind, I will join at once myself, and use any influence I may possess to induce some of my fellow-nurses to join also.

### From a Certificated Nurse and Diploma Midwife.

I must thank you for the weekly Hospital, which I have taken in since its commencement, and like very much. I gladly send you my name respecting the National Pension Fund for Nurses, being myself a certificated nurse and diploma midwife, working at present for a private nursing institution. I shall be glad to have full particulars from you respecting the Annuity Fund, as I know of both superintendents and nurses who are anxiously waiting to know the result before joining. As for myself, I have long and often been surprised that no such provision hitherto has been made for nurses when out of health, or when past work altogether. Nurses, as a rule, even the higher and better class of them, are often poor in this world's goods, and then their duties are most arduous sometimes both day and night; and shall I be allowed to say that nurses are often both in hospitals and private institutions underpaid for their labour? I am sure the Pension Fund would prove a great blessing to many a one who would be unable to work longer in the field, or even when requiring rest. I think if the Pension Fund was really made known to the public at large, many rich people would gladly give donations and subscriptions towards such a worthy object. I sincerely trust it will soon be established, and worked in such a way as to encourage all to join and receive a yearly pension.

### From the Matron of the Metropolitan Asylums Board Western Hospital.

Will you add my name to those already on the register as willing to join the Pension Fund, if it is successfully started, as one of our head nurses is also very anxious to join, and wants to know if there is any limit as to age, as she is forty-six? She has been trained, and been engaged in nursing since 1865. If you will let me know if she is eligible, I shall feel obliged. One of our nurses, to whom I spoke about joining the Fund, is also anxious to know if, after contributing for some time to the Fund, nurses getting married are barred from any participation in the benefits, or is their money returned?

### From a Nurse in a Nurses' Home.

Accept of my most sincere thanks for the great interest and trouble you have taken in the Nurses' Annuity Fund through The Hospital, which is a most delightful and useful paper. Myself and other nurses, whom I have spoken to about the Fund, think it a most excellent and needful thing. We do hope sufficient members will come forward, so that it may be *self*-supporting. We think it would be more generally liked. Please put my own and friends' name down on your list.

